# Procedures for Culturing and Genetically Manipulating Murine Hippocampal Postnatal Neurons

**DOI:** 10.3389/fnsyn.2020.00019

**Published:** 2020-04-30

**Authors:** Enora Moutin, Anne-Laure Hemonnot, Vincent Seube, Nathalie Linck, François Rassendren, Julie Perroy, Vincent Compan

**Affiliations:** ^1^Institut de Génomique Fonctionnelle (IGF), University of Montpellier, CNRS, INSERM, Montpellier, France; ^2^Laboratoire d’Excellence Canaux Ioniques d’Intérêt Thérapeutique (LabEx ICST), Montpellier, France

**Keywords:** neurons, postnatal, hippocampus, culture, transfection, transduction, lentivirus

## Abstract

Neuronal hippocampal cultures are simple and valuable models for studying neuronal function. While embryonic cultures are widely used for different applications, mouse postnatal cultures are still challenging, lack reproducibility and/or exhibit inappropriate neuronal activity. Yet, postnatal cultures have major advantages such as allowing genotyping of pups before culture and reducing the number of experimental animals. Herein we describe a simple and fast protocol for culturing and genetically manipulating hippocampal neurons from P0 to P3 mice. This protocol provides reproducible cultures exhibiting a consistent neuronal development, normal excitatory over inhibitory neuronal ratio and a physiological neuronal activity. We also describe simple and efficient procedures for genetic manipulation of neurons using transfection reagent or lentiviral particles. Overall, this method provides a detailed and validated protocol allowing to explore cellular mechanisms and neuronal activity in postnatal hippocampal neurons in culture.

## Introduction

The hippocampus and cortex are two brain areas extensively studied due to their implications in several important neuronal processes including cognition, learning, and memory. Over the past 10 years, major technical breakthrough facilitated *in vivo* studies of these two structures. Despite these advances, *in vitro* models remain the easiest to implement, and are relevant for many applications to study neuronal pathophysiology. In particular, primary neuronal culture is a powerful model to more easily manipulate and observe neurons. This simplified environment facilitates gene manipulation, time-lapse microscopy, electrophysiology and biochemistry, among others. Cultures of dissociated neurons were historically developed from embryonic rats (Banker and Cowan, 1977), but were limited to short term culture (<5 days) or required to co-culture dissociated cells with tissue explants. More complex neuronal culture protocols were later developed for mature neurons and long-term studies. A commonly used method is the ‘sandwich’ method which requires growing neurons on coverslips on top of a layer of glia cells [for a detailed protocol see Kaech and Banker (2006)]. This model provides cultures of almost pure neurons and is of particular interest to study interaction between astrocytes and neurons dissociated from two different mouse lines. Other approaches have been developed which consist of either growing neurons directly on a confluent glial cell layer or using glia-conditioned medium to maintain neurons in culture for a long period. Culturing neurons without the need of a feeder layer of glial cells was made possible through the formulation of a commercial specific media called Neurobasal (Brewer et al., 1993). It has been designed with optimized concentrations of each component to promote neuron survival and is lacking some excitatory amino acids that can be toxic for neurons. Supplemented with a serum free supplement called B27, Neurobasal is currently by far the most popular culture media for primary neurons.

Several postnatal culture protocols have been published to produce mouse primary neuronal culture from either very early stage after birth (Ahlemeyer and Baumgart-Vogt, 2005; Beaudoin et al., 2012; Kaar et al., 2017) or adult animals (Eide and McMurray, 2005; Peltier et al., 2010). Postnatal culture presents important advantages such as (i) reducing the number of experimental animals in agreement with the rule of the 3R, as only the pups required for the culture are sacrificed, (ii) genotyping of transgenic animals prior to the culture. Despite these benefits, postnatal culture is still underutilized mainly because of inconsistencies in culture quality compared to embryonic hippocampal dissociated cultures. One explanation for this discrepancy likely comes from the composition of culture media, such as Neurobasal-A, which contains high level of NMDA receptor co-agonist such as L-cysteine or glycine, which can lead to neurotoxicity particularly during long-term postnatal or adult culture (Hogins et al., 2011; Maggioni et al., 2015). Indeed, excitotoxicity increases with the age of culture and correlates with the maturation of neuronal connectivity and the parallel increase of NMDA receptor expression (Peterson et al., 1989; Mattson et al., 1991; Brewer et al., 2007). To overcome these problems, Bardy and colleagues developed a new medium called BrainPhys, which recapitulates the *in vivo* neuronal *milieu intérieur* by adjusting the concentrations of inorganic salts, neuroactive amino acids, and energetic substrates. This medium better supports important neuronal functions and improves physiological neuronal activity on iPSCs- or ESCs-derived human neurons (Bardy et al., 2015).

Here, we thought to develop an optimized culture protocol for postnatal hippocampal neurons. First, we settled a gentle and fast protocol for cell dissociation and plating which can be achieved in less than 2 h, thus improving neuronal survival. Next, we combined the advantage of Neurobasal-A and BrainPhys media for cell plating/growing and for culture maintenance, respectively. Our protocol leads to robust and reproducible hippocampal postnatal cultures that can be successfully prepared from P0 to P3 mice. These cultures present comparable ratios of inhibitory versus excitatory neurons like in embryonic cultures, and provide a neuronal network with a physiological neuronal activity. Finally, we described detailed protocols to produce lentiviral particles and genetically manipulate these cultures either by transient transfection or viral transduction. We also provide few examples of experiments that can be performed on such cultures to manipulate and study neurons.

## Materials and Equipments

### Animals

All animal procedures were conducted in accordance with the European Communities Council Directive and supervised by the IGF institute’s local Animal Welfare Unit (A34-172-41). Mouse pups were obtained from timed-pregnant C57Bl/6j mice. Mice were housed in harem breeding composed of one male and three females. Animals were maintained in a 12 h light/dark cycle (lights on from 7:30 am to 7:30 pm), in stable conditions of temperature (22°C) and humidity (60%). Food and water were available *ad libitum*.

### Lentiviral Vectors and Others Plasmids

Packaging plasmid pMD2. G and psPAX2 plasmids were a gift from Didier Trono (Addgene plasmids #12259 and #12260). pAAV-hSyn-VARNAM was a gift from Vincent Pieribone (Addgene plasmid #115554). DRH313: FCK-CheRiff-eGFP and DRH337: AAV-hsyn-CheRiff-eGFP were a gift from Adam Cohen (Addgene plasmids #51693 and #51697). For transgene expression, backbones of pWPT-GFAPprom-RCaMP2, pWPT-CaMKIIαprom-GCaMP6, pWPT-CaMKIIαprom-Venus-NR1A, pWPT-CaMKIIαprom-NLuc-YPet and pWPT-CaMKIIαprom-LIMK-NLuc-YPet plasmids were all derived from pWPT-GFP plasmid (Addgene plasmid #12255). These plasmids were produced by Gibson Assembly (New England Biolabs) after amplification by PCR of GFAP or CaMKIIα promoters and GCaMP6 calcium indicators (amplified from pGP-CMV-GCaMP6s, gift from Douglas Kim & GENIE Project, Addgene plasmid #40753). Others DNA sequences were produced by gene synthesis and sub-cloned in one of the plasmids mentioned above.

### Imaging of Dissection Steps and Culture Development

For dissection steps, images were taken using a Canon EOS100D camera with an EFS 18–55 mm objective. To follow the development of neurons during the 14 days of culturing (Figure 3A), cells were plated on Ibidi dishes (81166) and representative images were taken at each time point using an Axiovert 40 CFL microscope with a 10X/0.25 objective. At DIV 0 (Day *In Vitro*) images were taken 1 h after cell plating.

### Immunocytochemistry

Cell cultures were fixed with 4% paraformaldehyde in PBS solution for 10 min and then permeabilized and blocked with a 3% BSA, 0.1% Triton X-100, PBS solution for 1 h at room temperature. Cultures were then incubated with primary antibodies overnight at 4°C. After washes, cells were incubated with secondary antibodies for 1 h at room temperature, washed, mounted on slides and observed under an Axio-Imager Z1 microscope equipped with appropriate epifluorescence and filters (Carl Zeiss). Image quantification was performed using ImageJ software. Synaptic puncta were quantified using Synapse Counter plug-in for ImageJ on images acquired with a Plan-Apochromat 40X/0.95 objective.

### Time-Lapse Calcium and Voltage Imaging

GCaMP6 and VARNAM fluorescences were recorded using a Zeiss Observer Z1 Microscope. For GCaMP6 fluorescence, microscope was equipped with a Zeiss filterset38 (λex 470/40 – λem 525/50) and a HXP 120C XP Carl Zeiss bulb. For VARNAM fluorescence, microscope was equipped with a 607/70 nm Brightline filter and excitation was performed using 550/15 nm LED with an intensity of 18 mW/mm^2^. Neurons were maintained in ACSF medium (in mM): 140 NaCl, 2 CaCl_2_, 3 KCl, 10 Hepes, 10 D-Glucose. pH was adjusted to 7,4 with NaOH and osmolarity was adjusted to 315 mOsmol using NaCl. Stimulation of CheRiff was induced by LED at 440/20 nm with an intensity of 2 mW/mm^2^.

### Biochemistry and Synaptosome Preparation

Synaptosomes were prepared from DIV 14 cultures using Syn-PER Synaptic Protein Extraction Reagent (Thermo Fisher Scientific) according to manufacturer instructions; proteins were separated by electrophoresis using 4–12% Bis-Tris Plus Bolt Gels (Thermo Fisher Scientific) and transferred on nitrocellulose membrane (iBlot Transfer Stack from Thermo Fisher Scientific). A solution of PBS supplemented with 0,1% Tween and 5% non-fat milk was used to block the membrane and dilute primary and secondary antibodies.

### Electrophysiology

Postnatal hippocampal neurons were recorded in the whole-cell patch-clamp configuration. mEPSCs were recorded on DIV 13 to DIV 15 neurons, at room temperature with a holding potential of −60 mV. The patch pipettes had a resistance around 4 MΩ when filled with the following medium (in mM): 140 CsCl, 0.5 CaCl_2_, 20 EGTA, 10 Hepes, 10 D-Glucose, 2 ATP-Na_2_, with an osmolarity of 300 mOsm and pH 7.2. Neurons were perfused with the following external medium (in mM): 140 NaCl, 2 CaCl_2_, 3 KCl, 10 Hepes, 10 D-Glucose, 1.5 MgCl_2_, 0.01 glycine, 0.01 bicucullin, 0.0003 tetrodotoxin. Currents were recorded through an Axopatch 200B amplifier and digitized at 3 kHz. Electrophysiological data were analyzed using the Clampfit 10 software from Axon instruments (Molecular Devices).

### Step-by-Step Protocol

#### Primary Culture of Hippocampal Neurons From Newborn Mice (P0 to P3)

Before starting the coating and the dissection steps, disinfect all the equipment and the laminar culture hood with 70% ethanol. If possible, sterilize tools by autoclaving. Manipulate coverslips using sterile forceps. All solutions have to be sterile.

***Coating of Petri dishes and coverslips. Timing 1 h spread over 2 days, at least 1 day before culture.***

(1) Thaw a poly-L-ornithine aliquot and dilute it five times in water to get a final concentration of 0.03 mg/mL. Fill the dishes with enough poly-L-ornithine to fully cover the bottom of the dish. As a guideline, we routinely use 1 mL for a 35 mm dish and 50 μL for a well of a 96 well plate. When culturing neurons on coverslips, make sure to remove any bubble remaining between the coverslip and the bottom of the dish.
(2) Incubate overnight at 37°C. This step can be realized 3 days in advance (over the week-end), not earlier.
(3) Wash three times with PBS. Do not remove PBS before you are ready for step 4.
(4) Dilute laminin to get a final concentration of 1 μg/mL in Neurobasal-A (dilution 1/1,000) and fill the dishes immediately after removing PBS. Be sure to cover the bottom of the dish and make sure to remove any bubbles if coverslips are used.
(5) Incubate at 37°C for 4–12 h.

***Final settings for dissection, cell dissociation, and plating. Timing: 15 min, just before starting the culture.***

The following material is sufficient for one culture prepared for up to eight animals. If doing several cultures of different mouse genotypes in parallel or if culture required more than eight animals, number of material and aliquots described in steps 7 and 8 have to be changed accordingly.

(6) On a clean bench, prepare dissection tools: big and small scissors, 1 pair of forceps, 1 small spatula.
(7) In a culture hood, fill two 60 mm plates with 6 mL cold Hibernate (one to collect the brains and one to dissect hippocampi) and one 15 mL Falcon tube with 2 mL cold hibernate to collect hippocampi. Add 500 μL of Hibernate in a papain aliquot and prepare one syringe of 2 mL and a 0.22 μm filter for later.
(8) Set a water bath at 37°C and warm up the papain aliquot supplemented with Hibernate, the CM+ medium and a 4% BSA aliquot.
(9) If possible, place a binocular microscope in a vertical laminar flow hood. If not, dissection steps can be performed on a clean bench. Prepare small curved scissors and forceps close to the microscope.

**FIGURE 1:**
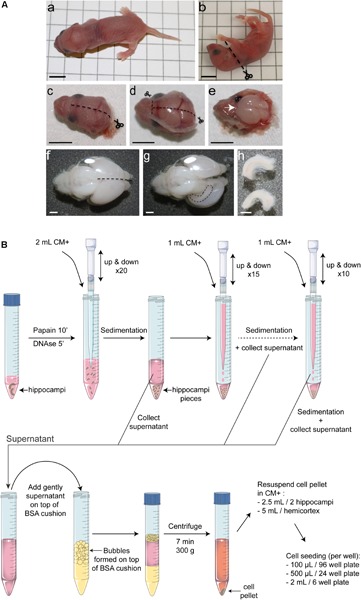
Overview of the protocol for mouse postnatal hippocampal culture. **(A)** Hippocampus dissection. After decapitation **(a–c)**, make a midline incision of the skin on the top of the head, from the back to the extreme rostral region of the brain. **(d)** Do exactly the same cutting along the skull moving forward slowly with the small scissors to avoid damaging the brain. Make two small incisions in direction of each eye and remove the skull. **(e)** Using a small spatula, gently remove the brain and place it into Hibernate medium. **(f)** Using small curved scissors, carefully separate the 2 hemispheres and ‘unroll’ the cortex from one hemisphere to make the hippocampus visible **(g)**. **(h)** Using small scissors remove the hippocampus. Black scale bar = 5 mm, white scale bar = 1 mm. **(B)** Schematic representation of the enzymatic and mechanical dissociation of cells and plating. Only two hippocampi are represented but this protocol can be applied for up to 16 hippocampi per tube. Hippocampi were collected in Hibernate-A medium, digested using papain at 37°C for 10 min and for five additional minutes in presence of DNase1. After stopping papain activity by adding CM+ media, the tissue was subjected to three rounds of mechanical dissociation using 1,250 μL filtered tips. After adding dissociated tissue on top of a 4% BSA cushion and centrifugation for 7 min at 300 *g*, cells were resuspended in CM+ media and seeded on pre-coated dishes.

**TABLE 1:** Reagents, consumables, dissection tools, and antibodies.

	**Designation**	**Supplier, reference**	**Comments**
Reagents	Antibiotics (penicillin and streptomycin)	Gibco, 15140-122	
	B27 supplement	Gibco, 17504044	
	Bovine serum albumin (BSA)	Sigma-Aldrich, A7906-50G	
	BrainPhys	Stemcell Technology, 05790	For culture maintenance
	Cytosine β-D-arabinofuranoside hydrochloride (AraC)	Sigma-Aldrich, C6645	To curb glia proliferation
	DMEM high glucose with Glutamax	Gibco, 61965058	
	DNase I	Roche, 10104159000	
	Fetal bovine serum (FBS)	Gibco, 10270105	Has to be heat inactivated for 30 min at 56°C prior to use
	Glutamax	Gibco, 35050037	
	Glutamine	Gibco, 25030-023	
	Hepes buffer 1M pH7.2 to 7.4	Gibco, 15630-055	
	Hibernate-A	Gibco, A12475-00	For culture dissection
	Laminin	Sigma-Aldrich, L2020	For coating
	Lipofectamine 2000	Invitrogen, 11668019	For transfection
	Neurobasal-A W/O Phenol red	Gibco, 12349015	For culture plating
	Papain	Sigma-Aldrich, P4761	For cellular dissociation
	Polyethylene glycol (PEG)	Sigma-Aldrich, 81253-250G	For lentiviral production
	Poly-L-ornithine hydrobromide	Sigma-Aldrich, P3654	For coating
Consumables	Conical tubes 15 mL	Falcon, CL-TFC55	
	Conical tubes 50 mL	Falcon, CL-TFC20	
	Filter (25 mm diameter, 0.2 μm)	Pall, CL-FS1	For papain filtration
	Filter (37 mm diameter, 0.2 μm)	Pall, CL-FS13	For CM filtration
	Filter (33 mm diameter, 0.45 μm)	Millipore, SLHV033RS	For lentiviral production
	Tips (with filter)	Sorenson, 34000	For physical dissociation
Dissection tools	Dumont #7 Forceps	FST, 11271-29	For dissection – steps 12, 15, 16
	Extra fine Bonn scissors straight	FST, 14084-07	For brain dissection – steps 11, 12
	Spatula	Dominique Dutscher, 037872	For brain removal – step 13
	Vannas spring scissors curved 4 mm cutting edge	FST, 15019-9	For hippocampus dissection – step 14
	Wagner scissors	FST, 14068-11	For pup decapitation – step 10
Primary antibodies	Actin	DSHB, JLA20	Dilution 1:2000 for WB
	CaMKIIα	Millipore, 05-532	Dilution 1:1000 for ICC
	Hoechst 33258	Sigma-Aldrich, B2883	Dilution at 1 μg/mL for ICC
	GFP	Biolabs,TP401	Dilution 1:2000 for ICC and WB
	GluN1	Synaptic systems, 114011	Dilution 1:1000 for WB
	GluR2	Sigma-Aldrich, MAB397	Dilution 1:1000 for WB
	MAP2	Sigma-Aldrich, M4403	Dilution 1:1000 for ICC
	NeuN	Synaptic Systems, 266006	Dilution 1:500 for ICC
	PSD95	ABR, MA1-045	Dilution 1:500 for ICC
	Prox1	Ozyme, BLE925201	Dilution 1:1000 for ICC
	RFP	MBL, PM005	Dilution 1:2000 for ICC and WB
	Synaptophysin	BD Biosciences, 611880	Dilution 1:1000 for ICC
	NeuN-A647	Abcam, Ab19565	Dilution 1:1000 for ICC
	GABA	Sigma, A2052	Dilution 1:2000 for ICC
	Homer1	Synaptic Systems, 160003	Dilution 1:1000 for ICC
Secondary antibodies	Donkey anti-mouse Alexa Fluor 488	Invitrogen, A21206	Dilution 1:1000
	Donkey anti-rabbit Alexa Fluor 350	Invitrogen, A10039	Dilution 1:1000
	Goat anti-chicken Alexa Fluor 594	Molecular Probes, A11012	Dilution 1:1000
	Goat anti-rabbit Alexa Fluor 488	Molecular Probes, A11034	Dilution 1:1000
	Goat anti-rabbit Cy3	Jackson Immunoresearch, 111-165-144	Dilution 1:1000
	Goat anti-mouse Cy3	Jackson Immunoresearch, 115-165-075	Dilution 1:1000

**Box 1. Preparations to be anticipated prior to postnatal hippocampal culture. In routine experiments, to perform the dissection and plating out neurons in less than 2 h, several preparations and aliquots have to be made in advance:**

– To avoid multiple freeze-thaw cycles and to save time, we strongly recommend to aliquot Cytosine β-D-arabinofuranoside hydrochloride AraC (10 mM), B27, glutamax, glutamine, heat inactivated FBS and antibiotics. All these reagents can be stored for months at −20°C.

– *Preparation of coverslips for immunocytochemistry. Timing 5 h, at least 2 day before culture.*

Clean coverslips can be prepared in advance and can be stored for months. Cell adhesion and polyaminoacids coating will be facilitated by using clean coverslips.

(1) Put coverslips in 1M HCl.

(2) Heat to 50–60°C for 4–8 h with occasional agitation.

(3) Wash the coverslips four times in milliQ water. Be sure to wash out the acid between stuck coverslips.

(4) Rinse coverslips in 100% ethanol.

(5) Store coverslips in 100% ethanol.

– *Preparation of Hibernate solution. Timing 5 min.*500 mL Hibernate bottle are supplemented with 5 mL antibiotics and can be stored for weeks at 4°C.

– *Preparation of 4% BSA aliquots. Timing 30 min. Can be stored at −20°C for months.*Dissolve 2 g of BSA in 50 mL of Hibernate, to get a final concentration at 4%. Filter using 0.2 μm pore size filter unit and aliquot by 2 mL in 15 mL tubes. Store at −20°C.

– *Preparation of DNase I aliquots. Timing 30 min. Can be stored at −20°C for months.*Resuspend DNase powder at 50 mg/mL in 50% glycerol, 2 mM CaCl_2_, 10 mM Tris pH 7.6, 50 mM NaCl. Always keep the DNase solution on ice during cell culture and put it back at −20°C after use.

– *Preparation of poly-L-ornithine aliquots. Timing 30 min. Can be stored at −20°C for months.*Dissolve 100 mg of poly-L-ornithine powder in 700 mL of milliQ water to get an intermediate concentration at 0.15 mg/mL and aliquot by 10 mL in 15 mL Falcon tubes. Store at −20°C.

– *Preparation of papain aliquots. Timing 30 min. Can be stored at −20°C for months.*On ice, dispense 2.5 mg of papain in 1.5 mL tubes. Store at −20°C. Papain will be freshly resuspended on the day of culture.

– *Preparation of culture media [supplemented (CM+) or not (CM−) with L-glutamine and FBS], see Table 2. Timing 30 min. Can be stored at 4°C for up to 2 weeks, to be filtered with 0,22 μm filter unit.*Depending on the number of pups used, adjust the quantity of media to prepare. Anticipate 3 mL of CM+ and CM− per pup.

***Removal of the brain. Timing 1–2 min for each pup.***

(10) Euthanize a mouse pup by decapitation using big scissors (Figure 1A-a,b) (see Table 1 for dissection tool references).
(11) By holding the head with the thumb on one side and the index on the other side, cut the skin of the head with small scissors from the caudal region to the extreme rostral region (dotted line on Figure 1A-c). Pull the skin on both sides; use the two same fingers to hold it down without pressing on the brain.
(12) Do exactly the same cutting along the skull moving forward slowly with the small scissors to avoid damaging the brain (Figure 1A-d). Between the two eyes, make two small incisions in direction of each eye and remove the skull using forceps. If the brain stays attached to the skull, gently insert the tip of the forceps in between and move back and forth.
(13) Place the tip of the spatula under the olfactive bulbs (Figure 1A-e) and use it to transfer the brain in the 60 mm dish containing cold Hibernate.
(14) Repeat steps 10 to 13 for each pup, pool the brains in Hibernate. Make sure that brains are all submerged in the Hibernate media.

**TABLE 2:** Preparation of culture media.

	**Components**	**Final concentration**	**Volume for 50 mL aliquot (in mL)**
Culture	Neurobasal-A	86.5%	43.250
medium	B27	2%	1
+ (CM+)	Glutamax	0.25%	0.125
	L-Glutamine	0.25% – 0.5 mM	0.125
	Antibiotics	1%	0.5
	FBS, heat inactivated	10%	5
Culture	BrainPhys	96.75%	48.375
medium	B27	2%	1
− (CM−)	Glutamax	0.25%	0.125
	Antibiotics	1%	0.5

***Dissection of the hippocampi. Timing 1–2 min for each pup.***

(15) Under the microscope, put one brain in a new 60 mm dish containing cold Hibernate. Hold the brain with forceps sunk in the cerebellum. Using small curved scissors, carefully separate the two hemispheres (dotted line on Figure 1A-f). Gently ‘unroll’ the cortex from one hemisphere to make the hippocampus visible. Using small scissors, remove one hippocampus (following the dotted line on Figure 1A-g). Repeat this step for the other hemisphere. You can eventually sink the forceps in the first hemisphere to get an easiest access to the other one.
(16) If meninges stay attached to the hippocampi, remove them using two pairs of forceps and transfer hippocampi in the 15 mL Falcon tube containing 2 mL of cold Hibernate (Figures 1A-h,B).
(17) Repeat steps 15 and 16 for each brain and pool hippocampi.

***Cellular dissociation and plating. Timing 1 h (Figure 1B).***

(18) Filter the 500 μL papain aliquot dissolved in Hibernate (step 7) using a 2 mL syringe and a small 0.22 μm filter. Start the dissociation by adding the papain solution to hippocampi. Incubate at 37°C for 10 min.
  Troubleshooting: do not use trypsin in place of papain otherwise it will result in an increase of cell death.
(19) Add 50 μL of DNase I. Dissociate tissue mechanically with a 1,250 μL filtered tip (5 up and down) and incubate 5 min at 37°C.
  Troubleshooting: the diameter of the tip is critical to get the expected dissociation. We routinely use Sorenson tips [1000 μl (50–1250 μl) XT Barrier – Catalog #34000].
(20) Stop papain activity by adding 2 mL of CM+ and dissociate tissue mechanically with a p1,000 pipette (20 up and down using 1,250 μL filtered tip). Let decant by gravity (1 min), collect supernatant in a new 15 mL Falcon tube and repeat dissociation two times on remaining debris (one with 15 up and down and one with 10) by adding 1 mL of CM+.
(21) Make a lot of bubbles (3 mL on graduations) in the 4% BSA aliquot (see Box 1), on top of which add delicately the supernatant.
(22) Centrifuge 7 min at 300 *g* at room temperature.
(23) Resuspend pellet in CM+. For a guideline, we resuspend two hippocampi in 2.5 mL of medium (corresponding to about 500.000 cells/mL).
(24) Remove laminin and directly plate the cells (100 μL for a 96 well plate, 500 μL for a 24 well plate, 1 mL for a 12 well plate and 2 mL for a 6 well plate).
(25) Keep the cultures in an incubator with constant temperature at 37°C and 5% CO_2_.

***Culture maintenance. Timing 1 h spread over 3 days.***

As a general rule, keep in mind that neuronal primary cultures are fragile. Minimize the time that the cells will spend outside the incubator.

**FIGURE 2:**
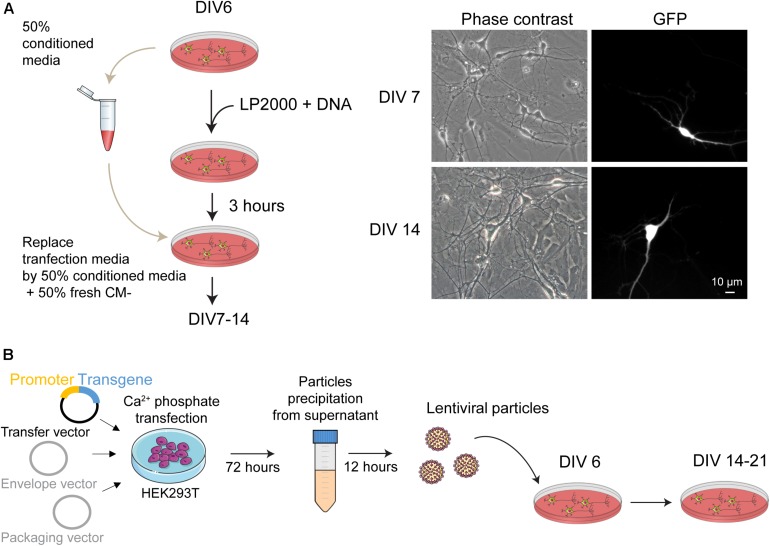
Genetic manipulation of mouse postnatal hippocampal culture. **(A)** (left panel) Schematic representation of the main steps for transgene expression in postnatal hippocampal culture using Lipofectamine2000 as transfection reagent. To reduce cellular toxicity, 3 h after the transfection the medium from the culture dishes is replaced by a solution composed by 50% of conditioned medium collected before the transfection and 50% of fresh CM– media. (right panel) Phase contrast and fluorescence picture of neurons 24 h and 8 days after transfection with a plasmid coding for the GFP protein. **(B)** Overview of the protocol used to produce and purify lentiviral particles from HEK293T cells. These particles are used to transduce the cultures at DIV 6. Expression of the transgene can be detected at DIV 14 and slightly increase until DIV 21.

**Box 2. Production of lentiviral particles (Figure 2B). Timing 4 h spread over 4 days with two waiting days in between. This protocol is adapted from Masuda et al. (2013). Before starting the lentiviral production, prepare the three following solutions that can be stored for months at 4°C:**

Solutions for calcium phosphate transfection of HEK293T cells

HBS 2X: to prepare 20 mL, mix 2.8 mL NaCl 2M, 2 mL Hepes 0.5M, 300 μL Na_2_HPO_4_ 0.1M and 14.9 mL milliQ water. Correct pH with NaOH and filter at 0.2 μm in a sterile hood. pH of the HBS solution will strongly influence transfection efficiency. We recommend to prepare four different HBS solution with pH ranging from 6.8 to 7.2. Test transfection efficiency using a plasmid coding for a fluorescent protein such as GFP to determine the optimal pH. If stored properly, HBS solution might be used for years without affecting transfection efficiency.

CaCl_2_ 1M: dissolve 14.7 g of CaCl_2_ in 100 mL milliQ water. Filter at 0.2 μm in a culture hood and aliquot per 10 mL. *Solution for lentivirus precipitation*Polyethylene glycol (PEG) solution 4X: to prepare 500 mL, mix 200 g PEG 6,000 with 100 mL milliQ water and agitate. Add 100 mL milliQ water and agitate. Add 40 mL NaCl 5M and agitate. Add 20 mL Hepes 1M pH 7.2–7.4 and agitate. Mix PEG, NaCl, and HEPES in this order or it may dissolve poorly. Adjust pH to 7.4 with NaOH and complete to 500 mL with milliQ water. Autoclave. (1) *Day 1: cell plating*Seed HEK293T cells in 10 140 mm Petri dishes at 30–40% density. Adjust final volume to 20 mL using fresh DMEM media (high glucose) supplemented with 1% antibiotics, 1% Glutamax, and 10% FBS. Depending on the quantity of virus to produce, this protocol can be up or downscaled to the appropriate quantity.Troubleshooting: use only HEK293T cells as this cell line contains the SV40 T-antigen and is competent to replicate vectors carrying the SV40 region of replication. Incubate the cells for 24 h at 37°C in a humidified incubator with an atmosphere of 5% CO_2_.

(2) *Day 2: phosphate calcium transfection*– Mix 200 μg of the expression vector plasmid, 50 μg of the packaging plasmid pMD2G and 150 μg of the envelope plasmid psPAX2 in a total volume of 8,250 μL of MilliQ water.

– Troubleshooting: only use plasmid DNA obtained from mid- or maxi-prep and resuspended at a concentration of at least 1 μg/μL.

– Add 2,880 μL of CaCl_2_ 1M and make bubbles during 30 s.

– Incubate the mixture for 20 min at room temperature.

– Add 11,520 μL of HBS 2X in a 50 mL Falcon tube.

– Add DNA mix to the HBS 2X tube drop by drop and make bubbles during 30 s.

– Immediately distribute the mixture in culture dishes (2.3 mL per dish).

– Between 6 and 8 h later, replace media by fresh DMEM (high glucose) supplemented with 1% antibiotics, 1% Glutamax, and only 1% FBS to reduce FBS precipitation.

Troubleshooting: before to replace transfection media, check that DNA precipitates are visible around cells using a 40X objective.

– Incubate 72 h at 37°C in a humidified incubator with an atmosphere of 5% CO_2_.

(3) *Days 5 and 6: lentivirus concentration by precipitation*– Filter the cell supernatant (containing lentiviral particles) through a 0.45 μm filter unit and distribute it in six 50 mL Falcon tubes (around 33 mL per tube).

Troubleshooting: filters get easily stuck so change it when pressure is too high; alternatively, centrifuge supernatant at 500 *g* before filtering in order to remove any floating cells and debris.

– Add one third volume of 4X PEG solution (around 11 mL per tube), mix by gently inverting five times.

– Incubate at 4°C overnight.

– Day 6: centrifuge the tubes at 2,600 *g* for 30 min at 4°C and discard the supernatant.

– On ice, resuspend the pellets in a final volume of 300 μL of PBS and make aliquots. Store at −80°C for years.

Troubleshooting: avoid introducing air bubbles when resuspending the virus to keep high transduction efficiency. Freeze-thaw of lentiviruses strongly impairs their efficiency so dispense into aliquots of appropriate volume.

(26) To limit glia proliferation, AraC treatment must be performed at DIV 2 during 8 to 18 h. Add AraC diluted in CM− (1 μM final dilution). As a guideline, we routinely dilute 1 μL of AraC 10 mM in 2 mL of CM− and then add 25 μL/well of 96 well plate, 120 μL/well of 24 well plate and 480 μL in a 35 mm dish.
(27) After AraC incubation, replace 80% of media by fresh CM− (80 μL/well of 96 well plate, 400 μL/well of 24 well plate and 1500 μL in a 35 mm dish).
(28) At DIV 6 or 7, proceed to a small replacement of culture medium. As a guideline, we routinely remove 300 μL of the culturing medium and add 400 μL of CM− for a 35 mm dish. If you plan to transfect or transduce the culture, skip this step and directly move to step 29.

#### Gene Expression

(29) At DIV 6–7, proceed to transfection with plasmid DNA or to transduction with AAV or lentiviruses. All the procedures have to be performed in a culture hood using sterile materials. All work using viruses must be performed after consulting your institution’s biosafety committee in order to determine the appropriate biosafety level.
  (29a) Transfection using Lipofectamine2000
    This protocol will lead to a strong and fast expression of the transgene into a low percentage of neurons (less than 5%, Figure 2A).
    – For the transfection of one 35 mm dish, prepare one tube with 250 μL Neurobasal-A + 2 to 4 μg plasmid DNA and one tube with 250 μL Neurobasal-A + 4 to 8 μL Lipofectamine2000. Wait for 5 min.
    – Pool the two tubes together, mix gently by doing few up and down and wait for 20 min.
    – Collect half of the medium from the culture dishes to be transfected and save it in a sterile tube. Keep this conditioned medium at 37°C. As a guideline, we routinely remove 1 mL from a 35 mm dish.
    – Carefully add the DNA/lipofectamine complexes drop by drop on the cells and put the culture dishes back in the incubator. Wait for 3 h.
    – Remove all the medium from the culture dishes and replace by a solution composed by 50% of the conditioned medium collected above and 50% of fresh CM−.
    – Expression of your transgene should be detected 24 h later.
  (29b) Transduction with lentiviral particles
    This protocol will lead to a low and slow expression of the transgene into a high percentage of neurons (close to 100% depending on the promoter used for transgene expression, Figures 2B, 5).
    – For a 35 mm dish, remove 300 μL of culture media and add a mixture of 400 μL of fresh CM− and 5 μL of lentiviral particles at 10^9^ IU/mL (see Box 2 for lentivirus production and purification). Amount of lentiviruses has to be adapted according to the transgene and the experiment to be performed. As a general guideline, we recommend to test three different concentrations of lentiviral particles to determine the optimal transduction when using a new viral construction.
    – There is no need to change media after lentiviral transduction.
    – Transgene expression will increase slowly and progressively with days. Depending on transgene and promotor activity, some expression might not be detected before 7 days.
  (29c) Transduction with AAV particles
    This protocol will lead to a high expression of the transgene into a high percentage of neurons (close to 100% depending on the promoter used for transgene expression).
    – For a 35 mm dish, remove 300 μL of culture media and add a mixture of 400 μL of fresh CM− and 0.1 μL of lentiviral particles at 10^11^ IU/mL. Amount of AAV has to be adapted according to the transgene and the experiment to be done. As a general guideline, we recommend to test three different concentrations of AAV particles to determine the optimal transduction when using a new viral construction.
    – There is no need to change media after AAV transduction.
    – Transgene expression will increase rapidly and progressively with days. Depending on transgene and promotor activity, experiments can be performed from the 3^*rd*^ days after the transduction.

## Results

### Development and Neuronal Activity of Mouse Postnatal Hippocampal Culture Maintained Either in BrainPhys or in Neurobasal Media

**FIGURE 3:**
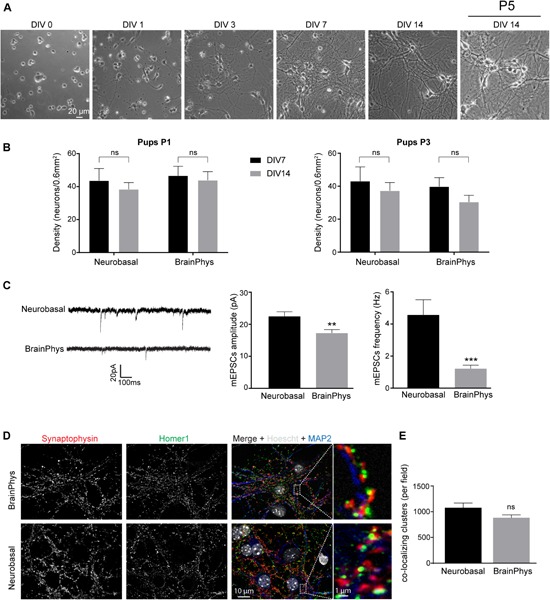
Neuronal morphology and activity in hippocampal culture growth in BrainPhys-containing CM– media. **(A)** Phase contrast images showing the development of a postnatal hippocampal culture. **(B)** Neuronal density of hippocampal culture from P1 (left panel) or P3 (right panel) pups growth in either Neurobasal-A or BrainPhys media. Cultures were fixed at DIV 7 or DIV 14 and neurons stained using NeuN antibody before quantification. Results represent mean + SEM, *N* = 3 independent cultures from P1 pups and P3 pups. At least 357 neurons were analyzed for each condition. Two way ANOVA corrected for multiple comparisons by controlling the False Discovery Rate (FDR). Ns indicates *q*-value > 0.05. **(C)** mEPSCs frequency and amplitude recorded between DIV 13 and 15 from hippocampal culture maintained in either Neurobasal-A or BrainPhys-containing CM–. Results represent mean + SEM, *N* = 27 and 31 cells were recorded for Neurobasal and BrainPhys condition respectively from four independent cultures done with pups aged between P1 and P2. Mann–Whitney test, **, *** indicate *p*-value < 0.01, *p*-value < 0.001, respectively. **(D)** Immunocytochemistry images from DIV 14 cultures stained with antibodies against the presynaptic marker Synaptophysin (Red), the dendritic marker MAP2 (Blue), the post-synaptic marker Homer1 (green) and Hoescht (Gray) in culture maintained in BrainPhys or Neurobasal-A medium. **(E)** Quantification of co-localizing puncta using the ImageJ plug-in called Synapse counter. For **(D,E)**, images were acquired using EC Plan-Neofluar 40X/1.30 and 100X/1.30 objectives respectively. For **(E)**, results represent mean + SEM, *n* ≥ 12 fields from *N* = 3 independent cultures from P1 pups. Unpaired *t*-test, ns indicates *p*-value > 0.05.

Currently most postnatal neuronal cultures are plated and kept in Neurobasal-A and/or DMEM media. Hippocampal cells were plated with CM+ because its rich composition in supplements might be important to promote cell adhesion and survival. After this initial phase, we thought that using BrainPhys medium (Bardy et al., 2015) instead of Neurobasal-A in the CM− would improve neuronal maintenance as this medium contains less neuroactive components that are likely detrimental for long term postnatal or mature neuronal cultures (Hogins et al., 2011; Maggioni et al., 2015). In embryonic neuronal culture such change in medium composition results in an improvement of action potential generation and synaptic communication and thus in the generation of an *in vitro* model closer to brain physiological conditions (Bardy et al., 2015). Before media change at DIV 3, neurons follow stereotypical developmental steps. First, after plating, neurons are spheres (Figure 3A-DIV 0) which start to acquire neurites at DIV 1. Between DIV 1 and DIV 4, these neurites grow and differentiate in axon and dendrites. We first compared basic properties of neurons kept after DIV 3, either in Neurobasal-A- or BrainPhys-containing medium. We did not observe any significant morphological differences between the two conditions of culture. The functional polarization begins approximately 1 week after seeding (DIV 7) with the formation of synapses. Between DIV 7 and DIV 14, a strong ramification of the dendritic tree is observed (Figure 3A-DIV 14) as well as the expression of synaptic markers (Figure 3D) consistent with the creation of a functional network. These experiments show that mouse postnatal hippocampal cultures kept in BrainPhys- or in Neurobasal-A-containing CM− undergo developmental stages which are similar to those previously described for embryonic culture (Meberg and Miller, 2003; Kaech and Banker, 2006). At DIV 7 and DIV 14, we did not observe any significant difference in neuronal density in cultures kept in Neurobasal-A or in BrainPhys (Figure 3B). Importantly, neuronal density was not affected by the age of mouse pups whatever the culture medium composition. Next, we studied the effect of both medium’s composition on neuronal excitability. Miniature excitatory post-synaptic currents (mEPSCs) recorded on neurons between DIV 13 and DIV 15 presented a significantly higher amplitude and a fourfold increase in the frequency when maintained in Neurobasal-A compared to BrainPhys (Figure 3C). These data support that the composition of culture media used for long term cell culture has important repercussions on neuronal activity. Cultures kept in Neurobasal-A are prone to hyperexcitability, whereas neurons kept in BrainPhys present spontaneous activity with features (amplitude and frequency of mEPSCs) close to what is observed *ex vivo* (Szczot et al., 2010; Thakar et al., 2017; Su et al., 2019). By co-immunostaining pre- and post-synaptic markers (Figure 3D), we detected presynaptic and post-synaptic protein clusters and quantify co-localizing puncta, which give an estimation of synaptic connections. We did not observe any significant difference in the number of co-localizing clusters (Figure 3E) between cultures maintained in Neurobasal-A and in BrainPhys suggesting that the difference observed in neuronal excitability is not caused by a change in synaptogenesis. We therefore decided to use BrainPhys medium for long term culture.

### Neuronal Composition of Mouse Postnatal Hippocampal Culture

**FIGURE 4:**
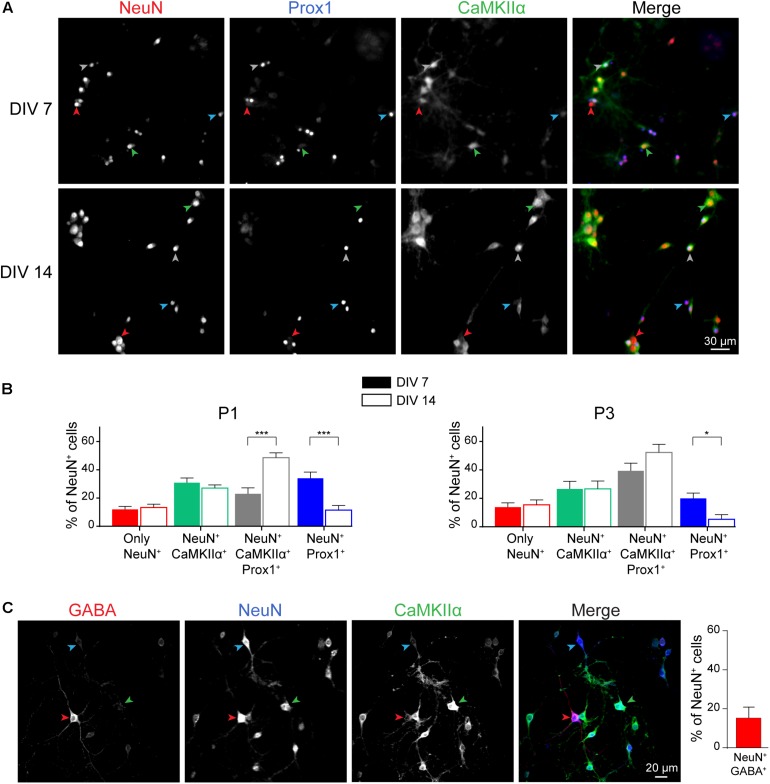
Neuronal cell type characterization of postnatal hippocampal culture. Immunocytochemistry images **(A)** and quantification **(B)** from DIV 7 and 14 cultures stained with antibodies against the neuronal markers NeuN (Red), Prox1 (Blue), and CaMKIIα (green) in culture maintained in BrainPhys medium. Red, Blue, Green and Gray arrow heads indicate cells only NeuN^+^, NeuN^+^/Prox1^+^, NeuN^+^/CaMKIIα^+^ and NeuN^+^/CaMKIIα^+^/Prox1^+^ respectively. For **(B)**, results represent mean + SEM, *n* ≥ 8 fields from *N* = 3 independent cultures from P1 pups and P3 pups. At least 357 neurons were analyzed for each condition. Two way ANOVA corrected for multiple comparisons by controlling the False Discovery Rate (FDR). *, *** indicate *q*-value < 0.05, *q*-value < 0.001, respectively. **(C)** Immunocytochemistry images from DIV 14 cultures stained with antibodies against the neuronal markers NeuN (Blue), GABA (Red), and CaMKIIα (Green) in culture maintained in BrainPhys medium. Blue, Red, and Green arrow heads indicate cells only NeuN^+^, NeuN^+^/GABA^+^, and NeuN^+^/CaMKIIα ^+^ respectively. Results represent mean + SEM, *N* = 3 independent cultures from P1 pups with *n* ≥ 4 fields per culture.

We next characterized the cellular composition of postnatal hippocampal culture. First, we analyzed the ratio of excitatory versus inhibitory neurons during culture development. Three main types of neurons are observed in the hippocampus: granular cells and pyramidal cells which are excitatory neurons, and inhibitory interneurons. We performed a triple co-immunostaining using (i) NeuN, a pan neuronal cell marker (Mullen et al., 1992; Iwano et al., 2012; Gusel’nikova and Korzhevskiy, 2015), (ii) CaMKIIα, a marker for hippocampal excitatory neurons (Benson et al., 1992; Sik et al., 1998; Wang et al., 2013; Pelkey et al., 2017), and (iii) Prox1, a granular cell marker (Iwano et al., 2012) (Figure 4A). All these markers are not necessarily exclusive and based on data from the literature, we considered that, (i) pyramidal cells are positive for NeuN and CaMKIIα, (ii) granule cells are cells positive for NeuN and Prox1 staining. Most of the granule cells, but not all, might be positive for CaMKIIα (Sola et al., 1999), (iii) inhibitory neurons are cells only positive for NeuN. Stainings were performed at DIV 7 (early stage of culture development) and DIV 14 (mature culture) on cultures from P1 and P3 mouse pups.

Whatever the age of pups, we observed that inhibitory interneurons account for ∼10–15% of the total neuronal cell population at DIV 14 (Figure 4B), while the remaining neurons are excitatory (85 to 90%), reflecting neuronal diversity observed *in vivo* in the mouse or rat hippocampus (Pelkey et al., 2017). Presence of inhibitory neurons was confirmed by performing immunostaining using anti-GABA antibody (Figure 4C). At DIV 14, GABA-positive and CaMKIIα-negative neurons represent 15,9 ± 5,4% of NeuN-positive neurons. Among excitatory neurons, proportion of granule and pyramidal cells was not affected as well by the age of pups (Figure 4B). Pyramidal neurons and granule cells represent around 30 and 60% of the NeuN positive cells, respectively. This is consistent with previous findings showing that postnatal hippocampal culture is mainly composed of granule cells (Wu et al., 2015).

During culture development from DIV 7 to DIV 14, proportion of Prox1^+^ granule cells, interneurons and pyramidal cells remain stable. However, we observed a decrease in the percentage of granule cells negative for CaMKIIα and a concomitant increase in the proportion of granule cells positive for this marker (Figure 4B). Similar observations were made on culture from P1 and P3 mouse pups.

At DIV 14 the postnatal hippocampal cultures are composed by roughly 50% of glial cells and 50% of neurons (data not shown), whatever the age of pups. Given the importance of glial cells and specially astrocytes in neurons homeostasis (Perea et al., 2009; Robertson, 2018), we believe that this heterogeneity in the culture is beneficial for neuron development, survival and activity as well as for providing an *in vitro* culture model closer to *in vivo* environment.

### Tools for Imaging Neuronal Functions

**FIGURE 5:**
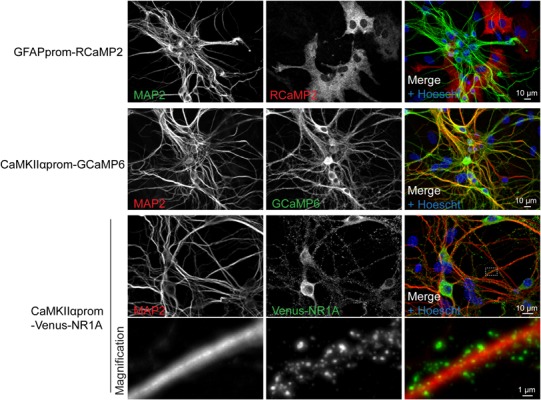
Lentiviral approach allows fine tuning, cell-type specific gene expression. Representative immunocytochemistry images realized on cultures transduced with two genetically encoded calcium indicators (RCaMP2 or GCaMP6) and the NMDA receptor subunit NR1A fused to Venus protein. These constructs were expressed under the control of promoters specific for either neurons (CaMKIIαprom) or astrocytes (GFAPprom). Neurons and nuclei were respectively stained with anti-MAP2 antibody and Hoescht, respectively.

Neuronal culture represents a model of choice to decipher neuronal cell biology at the molecular level, according that these cells are amendable to gene transfer. To that aim we developed a lentiviral approach that allows cell-specific gene expression. As illustrated in Figure 5, the present protocol for lentivirus production allows to efficiently genetically manipulate cells of postnatal cultures. We routinely expressed different genetically encoded calcium indicators either in astrocytes or in neurons using specific promoters (Figure 5). Lentiviral transduction allows low level of transgene expression levels without compromising efficiency in gene delivery. Such features enable a correct targeting of proteins of interest such as NMDA receptor subunit as demonstrated by the punctuate staining of Venus-NR1A subunit observed in dendrites (Figure 5).

**FIGURE 6:**
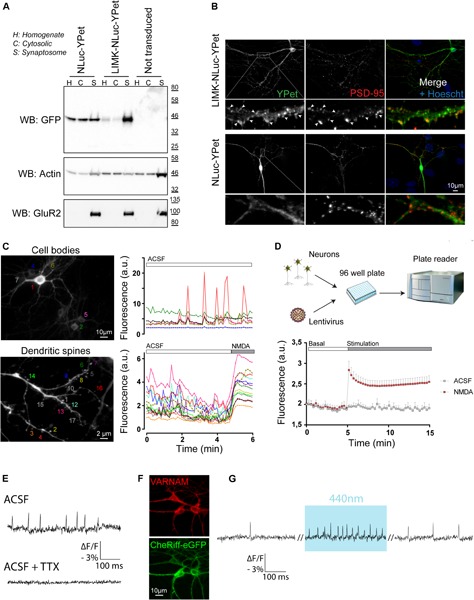
Postnatal hippocampal culture is a simple model to study neuronal function. **(A)** Identification of the subcellular localization of proteins in neurons. After synaptosome purification, expression of endogenous (actin and GluR2) and overexpressed proteins (NLuc-YPet and LIMK-NLuc-YPet) was evaluated by immunoblot in the homogenate (H), the cytosolic fraction (C) and synaptosomes (S). Note that the N-terminal tail of LIMK1 is sufficient to target the cytoplasmic construct NLuc-YPet to dendritic spines. **(B)** Identification of the subcellular localization of proteins in neurons by immunocytochemistry. NLuc-YPet constructs were detected using a GFP antibody recognizing YPet. Dendritic spines were stained using antibodies against PSD-95. **(C)** Calcium recording using videomicroscopy. Spontaneous fluorescence variations of neurons expressing GCaMP6 following lentiviral transduction were recorded for 6 min at 0.15 Hz. Fluorescence intensity was acquired in seven neuronal cell bodies (Top panels) and in 17 different dendritic spines before and after application of 50 μM NMDA (bottom panel). **(D)** Calcium recording using a plate reader. Postnatal culture was seeded in 96 well plate and transduced with lentiviruses coding for GCaMP6 at DIV 7 (top panel). Fluorescence was recorded using an Infinite F500 plate reader for 15 min before and after a control (ACSF) or 50 μM NMDA (NMDA) stimulation. **(E–G)** Monitoring and controlling neuronal activity by light. VARNAM, a red shifted voltage indicator was used to monitor spontaneous **(E)** and light-induced **(G)** neuronal activity at 600 Hz in cells co-expressing the blue shifted channelrhodopsin CheRiff **(F)**. Addition of 0.3 μM TTX completely abolished VARNAM -evoked fluorescence variations.

We usually performed experiments between DIV 14 to DIV 17 when culture is considered mature with a neuronal network composed of stabilized synapses, characterized by spontaneous activity. Results from Figure 6 highlight examples of experiments that can be achieved using our protocol for culturing and transducing postnatal hippocampal neurons using specific tools dedicated to study neuronal biology. First, to specifically target reporter proteins to spines, we used a palmitoylation motif of the N-terminal tail of LIMK1, as previously described (George et al., 2015). We generated LIMK-NLuc-YPet by fusing this LIMK1’s palmitoyl-motif with the N-terminal tail of NLuc-YPet, a fusion protein between the luciferase NLuc and a YFP variant. After transduction and expression in postnatal culture, we compared the subcellular localization of LIMK-NLuc-YPet with that of NLuc-YPet. Synaptosomes were purified at DIV 17 and the different fractions were analyzed by Western Blot. Results from Figure 6A show that NLuc-YPet construct is, as expected, indifferently detected in the homogenate, the cytoplasm and the synaptosomal fractions. On the opposite, when fused to the palmitoylation motif, LIMK-NLuc-YPet is preferentially detected in the dendritic spines. Internal controls show that the endogenous proteins actin and AMPA receptors (GluR2) are detected either in the three fractions or specifically in the synaptosomes, respectively. Preferential localization of LIMK-NLuc-YPet at the synapse was also confirmed by immunocytochemistry and co-staining with the post-synaptic marker PSD-95 (Figure 6B).

Primary cultures are also convenient for different kinds of functional experiments (Figures 6C–G). We first expressed the genetically encoded calcium reporter GCaMP6s under the control of the excitatory neuronal promoter CaMKIIα. By imaging fluorescence for 6 min, we detected spontaneous activity in individual neurons, either in cell bodies (Figure 6C, top panel) or in dendritic spines (Figure 6C, bottom panel). This experiment shows typical recording of neurons with spontaneous calcium waves either asynchronous or synchronous. On dendritic spines, application of 50 μM NMDA induced a robust and prolonged increase in calcium entry (Figure 6C, bottom panel). The present protocol for hippocampal postnatal culture is also compatible with 96 well-plate format and GCaMP6 fluorescence can be recorded on cell population using a plate reader (Figure 6D).

Second, we provide proof of principle that an all-optical approach can be used on postnatal cultures thereby allowing to optically monitor and control neuronal activity. In neurons after expression of the red shifted voltage indicator VARNAM (Kannan et al., 2018) using AAV-DJ particles, spontaneous action potentials (APs) were resolved by recording fluorescence at a frequency of 600 Hz (Figure 6E). Changes in fluorescence were completely abolished after application of TTX. In neurons co-expressing VARNAM and the blue shifted channelrhodopsin CheRiff (Figure 6F), light stimulation at 440 nm increased APs frequency (Figure 6G) confirming that neuronal activity can be controlled by light in cells expressing CheRiff and recorded through VARNAM fluorescence.

Altogether these data illustrate that our protocols provide an *in vitro* culture model for exploring cellular mechanisms and neuronal activity.

## Discussion

Postnatal hippocampal culture represents a convenient model for studying neuronal pathophysiology *in vitro*, especially when using transgenic animals. Most of the current procedures using new born mice are based on protocols originally developed for embryonic culture, resulting to inconsistency in culture quality or limited to P0-P1 pups.

Herein we thought to develop a protocol dedicated to postnatal culture by optimizing the two main steps of the culture, cell dissociation and cell feeding. First, by developing a fast and gentle protocol for cell dissociation we optimized the survival of hippocampal neurons collected from P0 to P3 mice. Contrary to numerous protocols designed for embryonic or postnatal cultures, we found that dissociation of hippocampi with the protease trypsin is detrimental by promoting neuronal death. In our hands, gentle enzymatic tissue digestion using papain is sufficient to dissociate cells preserving neuron survival. Similarly, reducing the mechanical stress by minimizing the trituration procedure results in a cell suspension with a high percentage of viable cells. Neuronal survival was also greatly improved during these different initial steps by keeping hippocampi and dissociated cells in Hibernate medium, a CO_2_-independent nutrient medium.

Second, we optimized culture conditions. Neurons are kept in Neurobasal-A medium until DIV 3 since its formulation was designed to favor cell attachment and survival following cell seeding. We found that using BrainPhys medium for long term cell culture results in neurons with reduced spontaneous activity compared to neurons kept in Neurobasal. In BrainPhys kept neurons, mEPSCs parameters are close to what is observed in hippocampal slices where network integrity is conserved (Szczot et al., 2010; Thakar et al., 2017; Su et al., 2019).

This decrease in hyperexcitability observed in BrainPhys is not associated with a change in neuronal survival or in the ratio between excitatory versus inhibitory neurons and is not caused by a delay in synaptogenesis. Indeed, whatever the culture medium used for cell maintenance, our results show that inhibitory neurons represent approximately 15% of the neuronal cells. We speculate that by providing a more physiological environment (Bardy et al., 2015), long term exposure to BrainPhys might regulate expression or post-translational modification of proteins involved in synaptic activity as already described for ARC protein in human neurons and thus facilitate neuronal maturation. This is supported by the observation of a switch of granular neurons from a negative to a positive staining for CaMKIIα during culture development. This switch reflects the development of granule cells toward a mature phenotype given that, (i) before P7, neurogenesis in the dentate gyrus is very active and GCs exhibit immature-like features (Ambrogini et al., 2004; Pedroni et al., 2014), (ii) in rat hippocampus, CaMKIIα mRNA expression increases during developmental stages and reaches a plateau in adult animals (Burgin et al., 1990) and, (iii) in adult mouse hippocampus where neurons are mature, the strongest expression of CaMKIIα is observed in the dentate gyrus (Wang et al., 2013). Thus, the change in CaMKIIα expression observed in our culture might reflect the progressive maturation of granule cells observed after birth.

Given the importance of glial cells in culture maturation and neuronal activity support (for a review see, Perea et al., 2009; Robertson, 2018), we designed a protocol using a low amount of AraC in order to limit rather than stopping glial cell development. In our experimental conditions, glial cells account for approximatively 50% of total cell number. Depending on the study to conduct, one might consider employing higher concentration of AraC to further limit glial cell proportion. However, it must be kept in mind that such treatment might impact neuron survival and/or affect synaptic development and neuronal activity.

We also describe two different approaches to genetically modify neuronal culture. The first method is based on transfection reagent such as Lipofectamine 2000. This approach is very simple to use, is very quick to set up and will result in an overexpression of the transgene. It represents an interesting strategy to express for instance a fluorescent marker in a small percentage of cells and visualize neuronal processing. While Lipofectamine 2000 might be toxic for neurons, we provide here a protocol that limits such toxicity. The second approach is based on the use of lentiviruses. Our protocol adapted from Masuda et al. (2013), is simple (no ultracentrifugation needed) and cost effective. It allows the production and the purification of lentiviral particles suitable for primary neuronal culture transduction. When combined with cell specific promoter, this strategy allows to target almost 100% of the desired cell type. In addition, because lentivirus lead to the integration of the transgene to the host genome, the use of appropriate promoters results in the expression level of exogenous genes closer to endogenous expression level, compared to other episomal approaches such as AAV transduction and transfection. Low expression level of transgene is illustrated by the correct localization of venus-NR1A subunit observed on Figure 5. Indeed, NR1 subunits require to co-assemble with others NMDA subunits to reach the cell surface (McIlhinney et al., 1996, 1998), supporting that in our experiments, expression level of venus-NR1A subunit is compatible with its association with endogenous NMDA subunits. In contrast, overexpression of NR1 tagged subunits using others approaches such as gene gun or transfection reagent result in retention of NR1 subunits and inappropriate localization in neurons (Hall and Soderling, 1997; McIlhinney et al., 1998; Barria and Malinow, 2002). We also routinely use AAV particles for gene manipulation in neurons. Expression of transgene is higher and faster compare to gene delivery using lentiviruses and can be detected 72 h following transduction depending on promoter activity. Purification of AAV particles is far more complex than that of lentiviruses or requires expensive commercial kit. Moreover, higher level transgene expression observed with AAV must be considered carefully including when using genetically encoded calcium indicator [for a review on impact of calcium sensor expression in neurons see McMahon and Jackson (2018)]. We believe that transgene expression using lentiviral particles represents by far the most effective and simple approach for genetic manipulation in culture.

This method for primary neuronal culture was originally designed for hippocampi from P0 to P3 pups. We found it can be extended up to P5 mice for hippocampal neurons without significant increase in neuronal death (Figure 3A). This flexibility in the age of pups extends the working time window and dispenses for precise breeding scheduling. This protocol can also be used for cortical neuron cultures from P0-P1 mice (data not shown). In this case, the use of older animals results in a decrease in neuronal survival and less reproducible cultures. Such cortical cultures are of particular interest when performing biochemical experiments where a large number of cells is required, since five 10 cm Petri dishes can be seeded from eight hemispheres (four pups).

Alternatively, this protocol can be adapted to 96 well-plates. Combined with our simple and cost-effective protocol for lentivirus production and neuronal transduction, this protocol of primary neuronal culture is suitable for experiments of automatized drug screening.

## Data Availability Statement

The raw data supporting the conclusions of this article will be made available by the authors, without undue reservation, to any qualified researcher.

## Ethics Statement

All animal procedures were conducted in accordance with the European Communities Council Directive and were reviewed and approved by the IGF institute’s local Animal Welfare Unit (A34-172-41).

## Author Contributions

VC and EM contributed conception and design of the study, performed the statistical analysis, and wrote the first draft of the manuscript. VC, EM, A-LH, NL, and VS performed the experiments. NL, A-LH, JP, and FR wrote sections of the manuscript. JP and FR critically revised the data. All authors contributed to manuscript revision, read and approved the submitted version.

## Conflict of Interest

The authors declare that the research was conducted in the absence of any commercial or financial relationships that could be construed as a potential conflict of interest.
